# High false discovery rate of the Architect anti‐HCV screening test in blood donors in Uganda and evaluation of an algorithm for confirmatory testing

**DOI:** 10.1111/vox.13364

**Published:** 2022-10-11

**Authors:** Olivia Lucey, Susan Acana, Peter Olupot‐Olupot, Rita Muhindo, Ronald Ayikobua, Sophie Uyoga, Dorothy Kyeyune‐Byabazaire, Graham Cooke, Kathryn Maitland

**Affiliations:** ^1^ Department of Infectious Disease, Division of Medicine Imperial College London UK; ^2^ Kilifi County Hospital and Kenya Medical Research Institute (KEMRI)‐Wellcome Trust Research Programme Kilifi Kenya; ^3^ Ugandan Blood Transfusion Service Kampala Uganda; ^4^ Busitema University Faculty of Health Sciences Mbale Campus and Mbale Regional Referral Hospital Mbale Uganda; ^5^ Department of Paediatrics Mbale Clinical Research Institute Mbale Uganda; ^6^ Institute of Global Health and Innovation, Division of Medicine Imperial College London UK

**Keywords:** blood transfusion services, donor blood, false positive, Hep C virus, screening, transfusion‐transmitted infections

## Abstract

**Background and Objectives:**

Adequate supplies of donor blood remain a major challenge in sub‐Saharan Africa. This is exacerbated by a lack of confirmatory testing for transfusion‐transmitted infections by blood transfusion services (BTS), leading to significant blood disposal owing to putatively high seroprevalence rates amongst Ugandan blood donors. We aimed to ascertain the false discovery rate of the Architect anti‐hepatitis C virus (HCV) screening assay and categorize screen‐reactive samples into three groups: presumed false positive, active and past infection, and develop an algorithm for confirmatory testing.

**Materials and Methods:**

A total of 470 screen‐reactive HCV blood donations were retested using the Architect anti‐HCV assay, an alternative antibody test (SD Biosensor) and a core antigen (cAg) test. signal‐to cut‐off (S/CO) ratios and pre‐analytical factors (centrifugation speed, haemolysis check, time between collection and testing) were recorded. Based on the S/CO ratio evaluation, we propose a testing algorithm to guide supplemental tests.

**Results:**

The false discovery rate of the Architect anti‐HCV assay was 0.84 as 395/470 (84%) screen‐reactive samples had no evidence of HCV infection (SD Biosensor and cAg negative) (presumed false positive), 38/470 (8.1%) were antigenaemic, and 32/470 (6.8%) had evidence of past infection. The median S/CO ratios of the presumed false‐positive and active infection samples were 1.8 and 17.3, respectively. The positive predictive value of HCV positivity in samples with ratios above 12 was 91.8%. On retesting, 104/470 (22.1%) samples became negative.

**Conclusion:**

The Architect anti‐HCV assay has a very high false discovery rate in Ugandan BTSs, leading to excessive blood disposal. Pre‐analytical factors likely contribute to this. An introduction of confirmatory testing using an algorithm based on S/CO ratio evaluation could limit unnecessary blood wastage and donor deferral.

**Highlights**

- Eighty‐four per cent of screen‐reactive hepatitis C virus (HCV) blood donations in Ugandan blood transfusion services were identified as presumed false positive.
- Active HCV infection is only present in 8.1% of screen‐reactive HCV samples.
- Introduction of confirmatory testing using an algorithm based on signal‐to cut‐off ratio evaluation could limit unnecessary blood wastage and donor deferral.

## INTRODUCTION

Providing safe blood for transfusion in sub‐Saharan Africa (sSA) remains a challenge in the context of limited resources and infrastructure, suboptimal diagnostics and a high prevalence of transfusion‐transmissible infections (TTIs) [1]. The epidemiology and true burden of hepatitis C virus (HCV) in sSA is unclear and overestimated due to prevalence estimates often relying solely on serology without confirmatory testing [2]. Furthermore, there is a substantial reported biological false‐positive rate in screening serology tests, particularly in sSA, where it is hypothesized that persistent exposure to infections, such as malaria, schistosomiasis, syphilis or HIV, as well as the presence of chronic disease and malnutrition may be exacerbating the issue [3, 4].

The mean HCV seroprevalence rate in blood donors attending Ugandan blood transfusion services (BTS) over the last 5 years is 1.8% (O. Lucey, personal communication); however, the rate ranges between 0% and 8% depending on geographical region and season. National HCV seroprevalence rates for the general population are estimated at 2.7% (0.4–7.0) [5, 6]. There is limited recent literature on rates in blood donors specifically [7]. The two‐step screening process for HCV, endorsed by CDC and WHO, involves the detection of antibodies against HCV (anti‐HCV) followed by either nucleic acid testing (NAT) or core antigen (cAg) testing to confirm the presence of active infection [8, 9, 10]. However, in Ugandan BTS centres, samples testing positive on the FDA‐approved Architect anti‐HCV screening assay (Abbott Diagnostics) do not undergo supplementary testing due to the lack of funding. Without the ability to confirm HCV infection status, the BTS are obliged to discard donations that are anti‐HCV initially reactive (IR) (regardless of duplicate testing results on the same assay). Reporting results without supplemental testing causes unnecessary anxiety for donors and reduces the donor pool due to donor deferral in the context of scarcity of blood for transfusion [11].

The barriers to the implementation of NAT‐based testing are well described and significant in low‐ and middle‐income countries (LMICs) [12]. HCV cAg testing is an alternative confirmatory test, which, although not extensively evaluated in blood donor populations, has proved an attractive alternative [13, 14, 15]. Unpublished data from retesting quarantined donor blood that was subsequently discarded by Mbale BTS (Eastern Uganda) during a multi‐site transfusion trial in Uganda showed that 45/50 (90%) of seropositive blood units were, in fact, RNA‐PCR negative, suggesting a high false‐positive rate in the Architect anti‐HCV screening assay. Sommese et al. and Candotti et al. also found 10% RNA positivity amongst seroreactive donor samples from anti‐HCV screening assays suggesting either high false‐positive rates or cleared infection [16, 17].

This study aimed to determine the false discovery rate of the anti‐HCV screening assay used in two Ugandan BTS centres by retesting screen‐reactive samples with supplemental tests. It aimed to understand the true prevalence of HCV amongst quarantined HCV screen‐reactive blood and categorize this into three groups: active HCV infection, past cleared infection and presumed false‐positive results. Secondary aims were to understand the significance of signal‐to‐cut‐off (S/CO) ratios of the Architect anti‐HCV assay, propose an HCV testing algorithm and investigate pre‐analytical factors affecting results, including haemolysis, time delay and centrifugation speed.

## METHODS

### Sample collection and storage

A total of 470 consecutive anti‐HCV reactive serum samples from voluntary non‐remunerated blood donors were identified between February 2019 and January 2020 in two BTS centres in Uganda: 235 from Nakasero National BTS, Kampala, and 235 from Mbale Regional BTS, Mbale. These samples had been collected, transported, and stored in the respective BTS laboratories, according to routine Ugandan BTS procedures. They had undergone TTI screening as per the National BTS algorithm by anti‐HCV chemiluminescent microparticle immunoassay (CMIA) on the Architect i2000SR analyser (Abbott Diagnostics, Germany) according to BTS Standard Operating Procedures that follow the manufacturer's instructions. A S/CO of ≥1.00 was considered IR and retested in duplicate as per the manufacturer's guidance. The S/CO ratio results for the first and duplicate runs for each sample were recorded directly from the automated system. Both IR and repeatedly reactive (RR) samples were included in the study and are termed ‘screen‐reactive’. Samples with S/CO ratios of 1.00–1.99 were described as borderline reactive in the analysis. A visual haemolysis check was performed using a colour chart on all samples as a proxy for sample quality. The date of blood collection and screening was documented. A delay between sample collection and testing was defined as more than 2 days. Screen‐reactive samples from both sites were prepared into serum aliquots for storage at −80°C. The 235 samples from Mbale underwent an extra centrifugation step (ultracentrifugation) using a microcentrifuge at 10,000 *g* for 10 min before storage to ascertain whether this affected the S/CO ratios on retesting.

### Supplementary testing

All 470 sera were thawed (after a single freeze–thaw cycle), mixed thoroughly by low‐speed vortexing and retested using the same Architect anti‐HCV assay. S/CO ratios of the repeat testing were recorded to observe any differences in S/CO ratios between the BTS screening and repeat testing, particularly following ultracentrifugation in the Mbale samples.

In order to discriminate presumed false‐positive from true‐positive results (both active infection and past infection), samples underwent two supplementary tests: an alternative antibody‐based test and a test to confirm active infection (HCV cAg). The antibody‐based test was the STANDARD Q HCV Ab Test (SD Biosensor, Korea), a rapid chromatographic immunoassay for the qualitative detection of specific antibodies to HCV, prequalified by WHO in 2020 with a quoted sensitivity and specificity of 99.4% (96.6–100) and 99.7% (98.3–100), respectively [18]. The Architect HCV Ag assay was performed on the automated Architect i2000SR CMIA system. The cut‐off value is 3.00 fmol/L; thus, samples with values of <3.00 fmol/L are considered non‐reactive, and those >10.00 fmol/L are considered reactive. Samples between 3.00 and 10.00 fmol/L are considered grey zone. These supplementary tests were performed (within 48 h of sample thaw) in the National BTS laboratory according to the manufacturer's instructions.

### HCV infection category definitions after analysis

All samples included in the study were screen‐reactive, that is, were either IR or RR on BTS Architect anti‐HCV screening. Samples also positive for HCV cAg were considered to represent active HCV infection (antigenaemic). Those negative for HCV cAg, with positive SD Biosensor results, were interpreted as past, resolved HCV infection. Samples negative for both SD Biosensor and HCV cAg were regarded as presumed false‐positive anti‐HCV results with no evidence of HCV infection. Samples that fell within the grey zone for the cAg assay or were invalid were categorized as unconfirmed.

### HCV testing algorithm

Based on S/CO ratio evaluation of the Architect anti‐HCV assay, we propose a testing algorithm to guide supplemental tests on IR samples. We applied our dataset to the proposed algorithm.

### Ethics

Ethical approval was sought and granted by the Imperial College and Ugandan Research Ethics Committees.

### Statistics

Statistical analysis was performed in GraphPad Prism version 9.0 (GraphPad Software, San Diego, CA, USA). Median with interquartile range (IQR) was used for S/CO ratios. Median differences in S/CO ratios between BTS screening and repeat testing were analysed using the Wilcoxon signed‐rank test. The presence of haemolysis by time delay between collection and testing was analysed using *χ* ^2^ for categorical variables. Significance was defined as a *p* value of <0.05.

## RESULTS

### HCV infection category results after analysis

**FIGURE 1:**
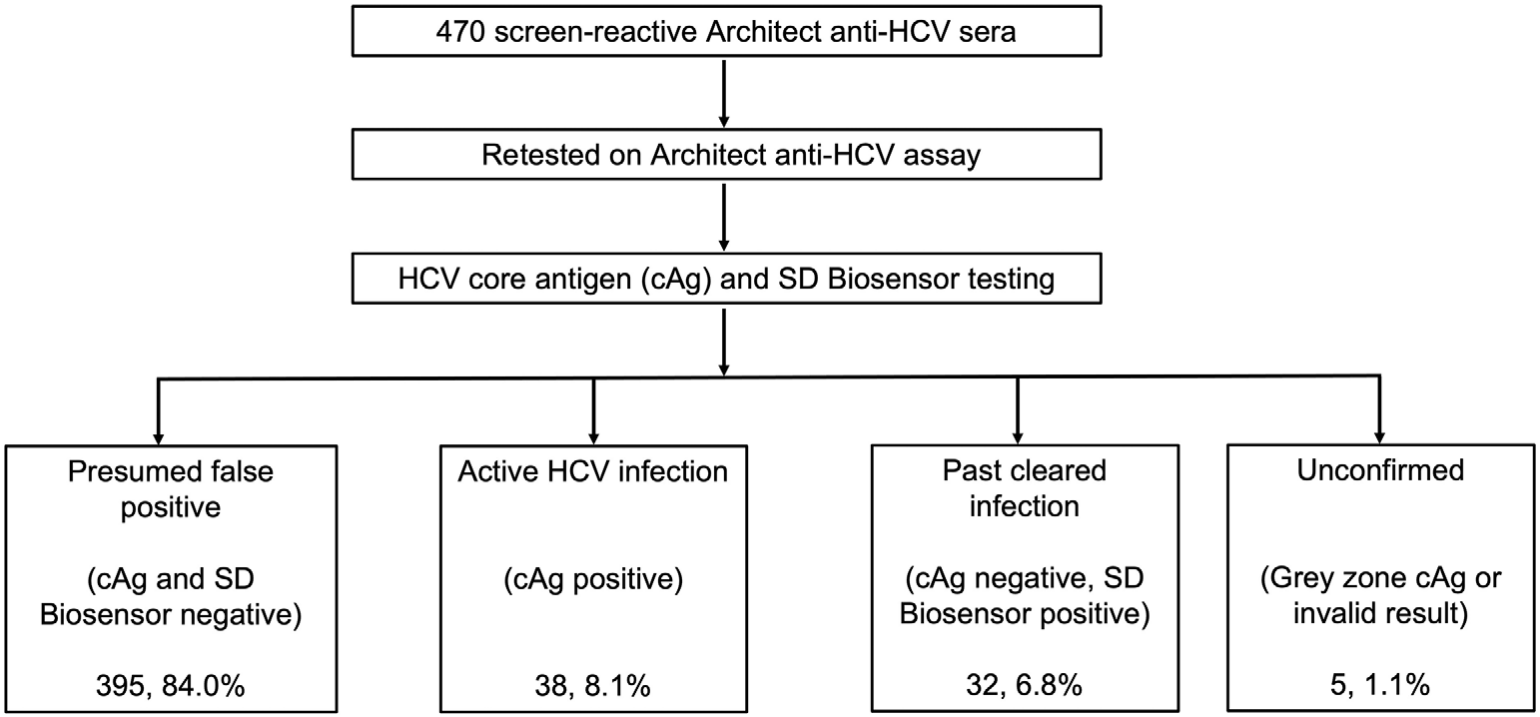
Hepatitis C virus (HCV) results following repeat (Architect anti‐HCV) and further (HCV core antigen and SD Biosensor) testing

Following anti‐HCV retesting and additional testing, 395/470 (84.0%) of the screen‐reactive samples tested negative by SD Biosensor and HCV cAg, confirming these as presumed false positives (Figure 1). In these two BTS centres, the false discovery rate of the screening Architect anti‐HCV test was, therefore, 0.84 (395/470), which translates to a positive predictive value of 0.16 (16%). Only 38/470 (8.1%) samples were antigenaemic with evidence of active HCV infection. A further 32/470 (6.8%) samples had evidence of past cleared HCV. Of the five unconfirmed samples, one had an invalid cAg result, and four had a cAg result between 3 and 10 f/mol (grey zone); two of which had positive SD Biosensor results, and two were negative. These were not retested in duplicate due to a lack of reagents and cannot be classified without further testing. Of the 444 samples across both sites, 433 (97.5%) were RR on the first BTS screening (26 samples did not have duplicate runs due to lack of reagent). Therefore, the calculated false discovery rate is estimated based on 470 screen‐reactive samples that included a small proportion (2.5%) of only IR samples.

### HCV results in relation to S/CO ratio

A S/CO ratio of >15 correlated with a positive cAg in 35/41 (85.4%) samples and a positive SD Biosensor result in 39/41 (95.1%) samples (Table 1). In the highest S/CO ratio category (>15), all 35 cAg‐positive samples also tested positive by SD Biosensor. Thirty‐six (94.7%) of the 38 positive cAg samples were samples with S/CO ratios of above 12. Thirty‐six (73.5%) of the 49 samples with S/CO ratio > 12 were cAg positive, and 45/49 (91.8%) were also positive by SD Biosensor.

**TABLE 1:** Additional hepatitis C virus (HCV) test results according to Architect anti‐HCV signal‐to‐cut‐off (S/CO) ratio following repeat and further testing

S/CO ratio	Total (*n*, %)	SD Biosensor positive		HCVc Ag negative (>10 fmol/L)	
<1.00	104 (22.1%)	1	1.0%	0	0.0%
1.00–1.99	121 (25.7%)	2	1.7%	1	0.8%
2.00–4.99	141 (30.0%)	10	7.1%	1	0.7%
5.00–9.99	46 (9.8%)	11	23.9%	0	0.0%
10.00–11.99	9 (1.9%)	1	11.1%	0	0.0%
12.00–14.99	8 (1.7%)	6	75.0%	1	12.5%
15–18.99	41 (8.7%)	39	95.1%	35	85.4%
Total	470	70		38	

In three (3/38; 7.9%) cAg‐positive samples, the SD Biosensor was negative, suggesting false‐negative SD Biosensor results. The anti‐HCV S/CO ratios of these three samples were 1.4, 3.1 and 17.5 (with cAg values of 500, 5043 and 690 fmol/L, respectively). Two (2/345; 0.6%) of the samples with S/CO ratios less than four had confirmed cAg presence. A significant proportion (113/134; 84.3%) of samples with S/CO ratios of 3.00–11.99 were classified as presumed false positives. The median S/CO ratio of the presumed false‐positive samples was 1.8 (IQR 1.0–3.2), whereas that of the cAg‐positive samples was 17.3 (IQR 16.3–18.1). The median ratio of the samples displaying past cleared infection was 5.7, with a wider IQR of 3.7–11.3.

### Pre‐analytical factors

On the first anti‐HCV screen, a large proportion of samples (256/470; 54.5%) were borderline reactive (S/CO ratios 1.00–1.99) (Table 2). One‐hundred and four (22.1%) of the 470 screen‐reactive samples became negative (S/CO ratio < 1.00) following retesting, with 73/104 (70.2%) of these occurring in samples from Mbale Regional BTS where an extra centrifugation step had occurred. To note, 7/104 (6.7%) of these samples were not RR on the first BTS screen. The median S/CO ratios of these samples on the first BTS screen were 1.22 and 1.98 in Nakasero and Mbale, respectively, with median decreases in S/CO ratios (between the first screen and retesting) of 1.42 in Mbale (*Z* = 7.42, *p* value > 0.001) and 0.41 in Nakasero (*Z* = 6.12, *p* value > 0.001). Following retesting of all samples, a statistically significant median increase in S/CO ratio of 0.45 was seen in all samples from Nakasero (*Z* = −8.00, *p* value > 0.001), whilst a non‐statistically significant median decrease of 0.15 was observed (*Z* = 1.48, *p* value = 0.14) in Mbale.

**TABLE 2:** Comparison of the distribution of signal‐to‐cut‐off (S/CO) ratios between blood transfusion services (BTS) Architect anti‐hepatitis C virus (HCV) screening and repeat Architect anti‐HCV testing

Architect anti‐HCV S/CO ratio	BTS screen‐reactive samples (*n*, %)	Retest (*n*, %)
<1.00	0 (0%)	104 (22.1%)
1.00–1.99	256 (54.5%)	121 (25.7%)
2.00–4.99	135 (28.7%)	141 (30.0%)
5.00–9.99	30 (6.4%)	46 (9.8%)
10.00–14.99	23 (4.9%)	17 (3.6%)
≥15.00	26 (5.5%)	41 (8.7%)
Total	470	470

Overall, 262/458 (57.2%) samples showed some level of visual haemolysis, with a higher proportion of Mbale samples showing evidence of haemolysis compared to Nakasero samples (95/235 [40.4%] vs. 167/223 [74.9%]). The median time between donor sample collection and initial screening was 4 days (range 0–33 days) across both sites. There was a significant difference in the proportion of samples with haemolysis in those that were processed after a delay (>2 days) (185/298; 62.1%) versus those that were processed without delay (0–2 days) (77/157; 49.0%) (*p* value = 0.0075).

### Testing algorithm

**FIGURE 2:**
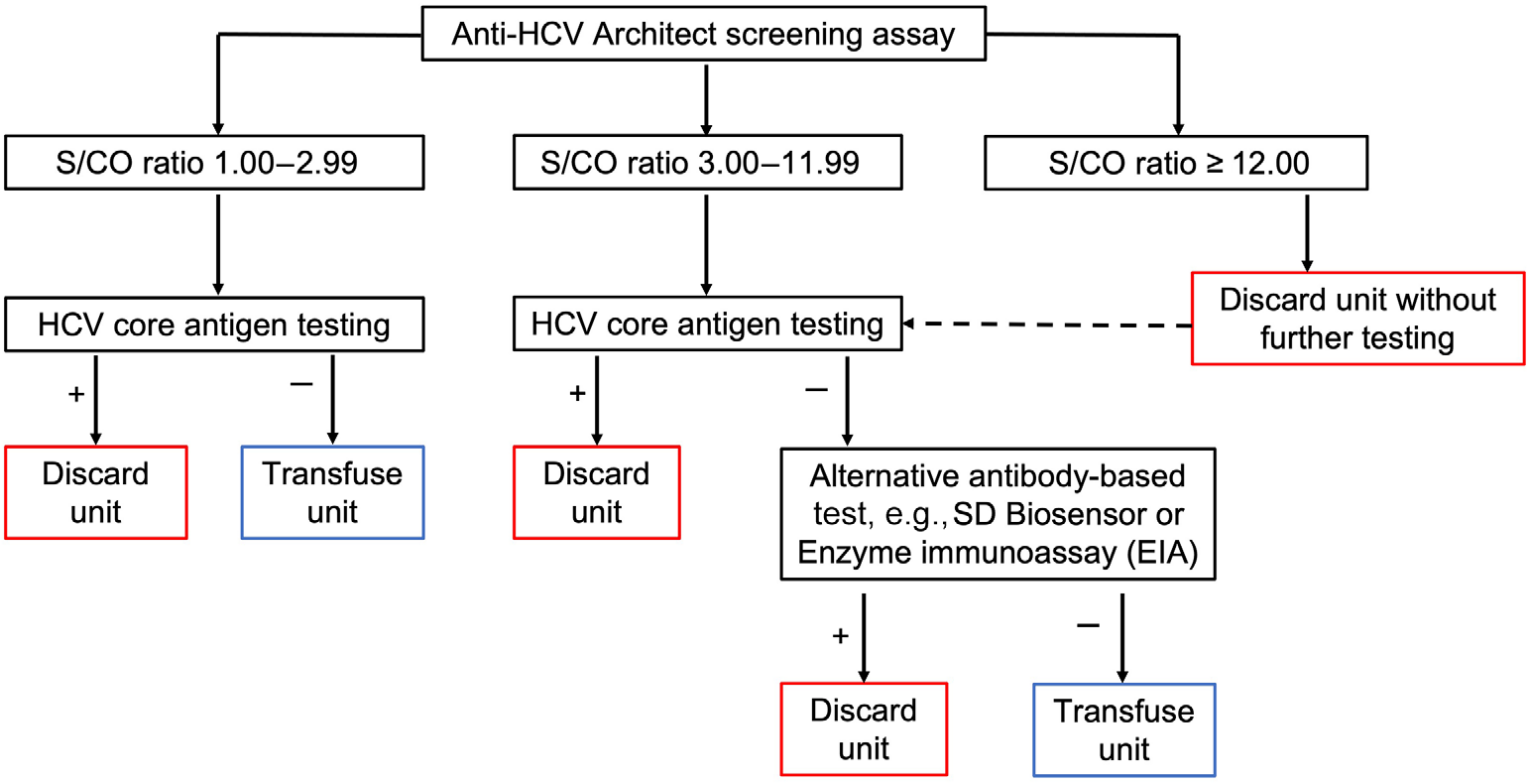
Proposed hepatitis C virus (HCV) confirmatory testing algorithm for initially reactive (IR) samples by Architect anti‐HCV assay. All IR samples with signal‐to‐cut‐off (S/CO) ratios between 1.00 and 11.99 would undergo core antigen (cAg) testing. Samples with low positive S/CO ratios (1.00–2.99) could be used for transfusion based on a negative cAg only, but samples with S/CO ratios between 3.00 and 11.99 would require an alternative antibody test if the cAg were negative

Figure 2 shows a proposed HCV testing algorithm based on S/CO ratios of the Architect anti‐HCV screening assay. Through the application of the algorithm to our dataset (Table 3), 317 cAg tests and 133 SD Biosensor tests would be required to ascertain whether the 366 reactive samples (S/CO ratio > 1.00 following repeat anti‐HCV testing) were confirmed positive or presumed false reactive. Of the samples, 70/366 (19.1%) units would have been discarded based on either a S/CO ratio > 12 or a positive cAg or SD Biosensor result amongst IR samples with a S/CO ratio between 3.00 and 11.99, 49/70 units (those with S/CO ratio > 12) would have been discarded without the need for any supplemental testing, 45/49 of these had either active or past infection (Table 1). Following supplemental tests, 296/366 (80.9%) units would have been recommended for transfusion following supplemental tests, including five anti‐HCV positive, cAg‐negative (past infection) samples in the 1.00–2.99 category. No cAg‐positive samples would have been recommended for transfusion.

**TABLE 3:** Application of proposed hepatitis C virus (HCV) testing algorithm to our dataset

Architect anti‐HCV S/CO ratio after analysis	Total no. of samples (366)	No. of core antigen (cAg) tests (positive/total performed)	No. of SD Biosensor tests (positive/total performed)	No. of incorrectly identified samples (*n*, %)	No. of units recommended for transfusion (*n*, %)
1.00–2.99	183	1/183	NA	5/183 (2.7%)^a^	182/183 (99.5%)
3.00–11.99	134	1/134	19/133	0	114/134 (85.1%)
≥12	49	NA	NA	4/49 (8.2%)^b^	0/49 (0%)

*Note*: Total number of samples is 366, as 104 samples became negative after retesting. NA: not applicable according to the algorithm.

Abbreviation: S/CO, signal‐to‐cut‐off.

^a^ Cleared, past infection (cAg negative, SD Biosensor positive).

^b^ Presumed false positive (cAg negative, SD Biosensor negative).

## DISCUSSION

This study has shown the Architect anti‐HCV assay to have an alarmingly high false discovery rate in the context of a BTS laboratory in sSA. This, coupled with the lack of supplemental or confirmatory testing, has led to significant blood wastage. Our data confirm that 395/470 blood units (84%) IR for HCV were discarded unnecessarily. The positive predictive value (PPV) of 0.16 is unacceptably low for a screening test. The BTS report that each year, on average, 224,350 units are donated and screened, with 4038 units discarded on the basis of Architect anti‐HCV positivity (O. Lucey, personal communication). Assuming 84% of these are, in fact, false positive, an estimated 3392 units are discarded unnecessarily each year. Many of these donors were therefore preliminarily classified as HCV seropositive necessitating post‐donation counselling, retesting and donor deferral at a significant economic and personal cost to the BTS and donor, respectively.

False positivity in anti‐HCV screening immunoassays is well described and multi‐factorial, with reported false positivity rates of between 10% and 60% [19, 20]. Populations with a lower prevalence of HCV such as blood donors are more affected by false positivity [20, 21]. The need for high sensitivity in screening assays can come at a cost in terms of specificity, although CMIA is reported to have better specificity than enzyme immunoassays (EIAs) [22, 23]. There remains uncertainty in how to interpret anti‐HCV test results with a borderline S/CO ratio, particularly without confirmatory testing [24]. The lower PPV of the Architect anti‐HCV assay discovered in this study compared to existing literature may reflect pre‐analytical factors in the context of a BTS rather than a research laboratory [20, 23, 25]. We suggest this may be more generalizable to other LMIC settings where transport delays, inadequate funding and laboratory infrastructure pose greater challenges than in high‐income countries and may affect test results. We demonstrated that 22% (104/470) of samples were anti‐HCV negative upon repeat testing following sample thaw. The extra centrifugation process in Mbale may have contributed to the difference in S/CO ratios seen between the first screen and retesting in this site, although the reduction was only statistically significant in the samples that became negative. Samples screened after a delay of more than 2 days were more likely to be haemolysed. We acknowledge that additional factors contributing to the S/CO ratio differences by site may have been present, for example, differences in sample collection and handling, including the presence of haemolysis, centrifugation equipment and differences in donor populations, for example, prevalence of chronic or infectious diseases [3, 4]. These pre‐analytical factors are likely contributing to the high false‐positive rate and should be further investigated and monitored through regular laboratory quality assessment, but achieving ‘perfect’ conditions can be extremely challenging in sSA [26].

Studies have shown a relationship between the S/CO ratio of the anti‐HCV test and the infection status of the individual [14, 16, 19, 27]. There is a correlation between high S/CO values and serological confirmation with anti‐HCV positivity, and in some studies, also with viraemia [23]. However, false positives and cleared infections are difficult to distinguish as both may have low S/CO values. Our data show that donors with active infection (cAg positive) had samples with median S/CO values that were almost 10‐fold higher than the presumed false positives (cAg and SD Biosensor negative). Our results are in keeping with other studies, which demonstrated significant differences in median S/CO ratios between RNA‐negative and RNA‐positive results [20] and cAg positivity rates of 83.6% in samples with S/CO ratios above 10 [14]. Knowledge of threshold ratios can help guide whether supplemental testing is required [28]. Ha et al. found that using logistic regression, a S/CO value threshold of 3.13 on Architect predicted HCV positivity (defined as anti‐HCV or RNA positive) with 95% probability, suggesting that samples with ratios above this could be reported as positive without supplemental testing. Other studies have reported 5.0 or 5.6 as the optimal cut‐off threshold based on the receiver operator characteristic curve analysis [20, 21, 28, 29]. Applying a cut‐off threshold of 3.13 to our samples would result in 110 samples (presumed false positives) being discarded unnecessarily. Optimal threshold ratios differ depending on HCV prevalence and laboratory settings. By setting the threshold at 12 in our dataset, the PPV of anti‐HCV positivity is much higher (45/49; 91.8%) and prevents unnecessary disposal of units.

We believe that our proposed testing algorithm based on S/CO ratio evaluation presents a pragmatic approach to providing safe blood whilst maintaining judicial use of supplemental tests in LMICs. Importantly, when applied to our dataset, no cAg‐positive samples would have been transfused, and only four false reactive samples (in the S/CO ratio > 12 groups) would have been discarded. The main drawback of omitting confirmatory tests in samples with higher S/CO ratios is that donors would not receive comprehensive results on infection status. By limiting the use of alternative antibody tests to samples with ratios of 3.00–11.99, there is a small but appreciable risk that samples in the lowest ratio category (1.00–2.99) may be anti‐HCV positive (cAg negative) suggesting past cleared infection. These donors would not be accepted for donation in Uganda under current guidelines. We identified 5/183 (2.7%) antibody‐positive cAg‐negative samples in the 1.00–2.99 group, which would have been recommended for transfusion. The risks of transfusing antibody‐positive blood would need to be balanced against resource scarcity, and we acknowledge this as a controversial area. One case of active infection occurred in a sample with a borderline reactive anti‐HCV screening test (S/CO ratio 1.4). This can occur during acute HCV seroconversion or in immunocompromised hosts [14], and strengthens the argument for confirmatory testing. The current regulatory requirement in Uganda to discard screen‐reactive samples due to a lack of confirmatory testing would need to be reviewed to enable the implementation of the algorithm.

Currently, all donors with screen‐reactive (IR or RR) results are deferred, either temporarily or permanently, depending on repeat testing results 3–6 months later and the availability of an alternative anti‐HCV test. The cost of permanent deferral is ~$81 (O. Lucey, personal communication from UBTS) and includes phlebotomy, initial TTI screening, retesting, post‐donation counselling and blood unit disposal. Using the proposed algorithm with supplemental tests (at $10/cAg test and $1/SD Biosensor) to confirm or refute positivity for the 366 donor samples was $1647 compared to the potential cost of permanent deferral of $29,646. In addition, accurate results through confirmatory testing would facilitate linkage to care where appropriate, avoid unnecessary anxiety and return visits for donors and enable those testing falsely positive to return to the donor pool. Furthermore, incorrect seroprevalence data generated as a result of poor diagnostics have repercussions on the accuracy of HCV epidemiology, surveillance, and linkage to care, and therefore, elimination strategies [5, 21, 30].

As described above, both a strength and limitation of this study is that screen‐reactive samples were identified for inclusion after BTS screening; therefore, the process by which they were taken and screened was not according to a research protocol, as such, samples were potentially subject to pre‐analytical errors such as sample haemolysis and testing delays. This may have affected the validity of the assays used in both BTS screening and subsequent testing. It may be a contributing factor to the high false discovery rate. We argue that it is important to present real‐world data in an effort to understand how tests are performing in LMIC laboratory conditions, and therefore, how to improve processes and tailor algorithms appropriately.

Our results and the proposed algorithm rely on the alternative antibody test possessing a similar sensitivity to the Architect anti‐HCV assay to reliably categorize screen‐reactive samples as either false positive or past infection. A recent large meta‐analysis showed that RDTs showed comparable sensitivity and specificity profiles compared to EIAs in diverse populations [31], and specifically, SD Biosensor achieved a sensitivity of 99.2% in a large sample size [32]. We acknowledge that the use of a rapid test, albeit a WHO prequalified test with comparable quoted sensitivity to Architect anti‐HCV, is not considered a gold standard test to detect anti‐HCV. Although using a combination of alternative antibody assays, including two EIAs and immunoblot testing, would achieve greater certainty in categorizing the anti‐HCV positive samples, this would not be financially or logistically feasible within the structure of Ugandan BTS. Three samples were falsely negative by SD Biosensor, suggesting that the test may have a poorer sensitivity compared to the Architect, assay or may be affected by genotypic differences or pre‐analytical factors [32].

The gold standard test to detect HCV RNA is NAT. We chose HCV cAg as the confirmatory test as NAT requires a dedicated sample to be taken to maintain RNA integrity, which was not feasible in this study. It is, therefore, conceivable that due to the slightly higher limit of detection of cAg, active infections could theoretically be missed. However, cAg testing is endorsed by WHO as a confirmatory test [8] and has several advantages over NAT; namely, it is less expensive, can be used on the existing i2000SR analyser, demands less personnel expertise and is less susceptible to contamination and degradation of samples [14]. Moreover, it has been shown to have good diagnostic accuracy and correlation with RNA, and although the analytical sensitivity is lower than NAT, more than 90% of patients with HCV have viral loads above 3000 IU/ml [5], making it an affordable, appropriate and an excellent alternative to NAT in LMIC settings where the realistic alternative at present is no confirmatory test.

In conclusion, due to both a high false discovery rate of the Architect anti‐HCV screening assay in this setting and the lack of access to confirmatory testing, a significant amount of blood donated is discarded unnecessarily. Since blood for transfusion is relatively scarce, focusing available resources on obtaining accurate HCV results would result in economic gains from limiting donor deferral and reducing unnecessary blood disposal. Optimization of pre‐analytical factors, including introducing ultra‐centrifugation before testing may reduce false‐positive rates, especially in samples with low positive S/CO ratios. Knowledge of Architect threshold S/CO ratios in predicting anti‐HCV positivity may be helpful in updating testing algorithms and guiding the supplemental tests with which to perform in screen‐reactive samples. We suggest that the algorithm we developed should be validated in other sSA BTS where HCV detection is higher than anticipated in low‐risk blood donor populations. The introduction of confirmatory testing in BTS settings is paramount for accurately identifying HCV‐positive donors who require follow‐up care and confidently distinguishing false‐positive and true‐positive anti‐HCV results to limit blood wastage and unnecessary donor deferrals.

## CONFLICT OF INTEREST

The authors declare no conflicts of interest.
